# A Comparative Analysis of Gene-Expression Data of Multiple Cancer Types

**DOI:** 10.1371/journal.pone.0013696

**Published:** 2010-10-27

**Authors:** Kun Xu, Juan Cui, Victor Olman, Qing Yang, David Puett, Ying Xu

**Affiliations:** 1 Department of Biochemistry and Molecular Biology, Institute of Bioinformatics, University of Georgia, Athens, Georgia, United States of America; 2 Department of Statistics, University of Georgia, Athens, Georgia, United States of America; 3 Joint Center for Systems Biology, Jilin University, Changchun, China; 4 College of Computer Science and Technology, Jilin University, Changchun, China; Dana-Farber Cancer Institute, United States of America

## Abstract

A comparative study of public gene-expression data of seven types of cancers (breast, colon, kidney, lung, pancreatic, prostate and stomach cancers) was conducted with the aim of deriving marker genes, along with associated pathways, that are either common to multiple types of cancers or specific to individual cancers. The analysis results indicate that (a) each of the seven cancer types can be distinguished from its corresponding control tissue based on the expression patterns of a small number of genes, e.g., 2, 3 or 4; (b) the expression patterns of some genes can distinguish multiple cancer types from their corresponding control tissues, potentially serving as general markers for all or some groups of cancers; (c) the proteins encoded by some of these genes are predicted to be blood secretory, thus providing potential cancer markers in blood; (d) the numbers of differentially expressed genes across different cancer types in comparison with their control tissues correlate well with the five-year survival rates associated with the individual cancers; and (e) some metabolic and signaling pathways are abnormally activated or deactivated across all cancer types, while other pathways are more specific to certain cancers or groups of cancers. The novel findings of this study offer considerable insight into these seven cancer types and have the potential to provide exciting new directions for diagnostic and therapeutic development.

## Introduction

Cancer is a key threat to people's health and life, accounting for ∼13% of all disease-causing deaths in the world [1]. In 2007, 7.6 million people died of cancer world-wide. In the U.S, over 1.4 million new cancer cases were reported each year in the past few years, and cancer becomes the second leading cause of death following heart disease. Statistics from the SEER reports indicate that the mortality rate across all cancer types in the U.S. went from 195.4 per 100,000 cases in 1950, continued an upward trend till 1978 reaching 204.4, and then steadily decreased to 184.0 in 2005 [2]. This decreasing trend has been mostly due to the improved diagnostic techniques for detecting the early stage of cancer. General survival statistics of cancer indicate that early detection and treatment are the key to longer survival across all cancer types.

Challenges in early cancer detection arise mainly from the reality that most patients are asymptomatic in the early stages of cancer, and only a few effective cancer-screening tests are clinically available. While some tests have proved to be effective in detecting cancer at its early stage, they are often too invasive, such as colonoscopy, to be routinely used during regular physicals and are currently limited to only a small number of cancer types. Often a cancer is already in an advanced stage when diagnosed; clearly, more effective techniques for early cancer detection are needed.

A number of genetic markers have been proposed for various cancers, such as BRCA1 and BRCA2 for breast cancer and CDH1 for gastric cancer. In addition, a number of promising serum markers for cancer have been used clinically. Among them, PSA (prostate-specific antigen) is the most well known and has been widely used for diagnosing prostate cancer through blood tests [3]. However, its effectiveness of detection is far from adequate, widely considered as having a false positive rate that is too high to be a reliable cancer-indicator [4]. Similar observations have been made about other serum markers such as CA125 for ovarian cancer [5].

Herein we present a computational study on prediction of both genetic and serum markers for seven cancer types, based on public microarray gene-expression data and a computer program for prediction of blood-secretory proteins [6]. Compared to earlier studies on cancer marker identification, including meta-analyses on multi-types of cancers [7], the present study has the following unique features: (i) a focus on identification of multi-gene markers through exhaustive analysis of all possible combinations of genes, taking full advantage of the available high-level computing power, rather than using heuristic approaches that may not necessarily find the optimal markers; (ii) an attempt to find markers for groups of cancers in addition to those for individual cancers; (iii) an attempt to link the information derived from transcriptomic data of tissues to marker prediction in serum using the novel prediction program [6]; and (iv) identification of pathways that are abnormally regulated, either common across multiple cancer types or specific to individual cancer types. We believe that these novel data will prove highly valuable in elucidating the genetic alterations in various cancers, as well as offering potential directions for new approaches in diagnostics and therapeutics.

## Materials and Methods

### 1. Microarray gene expression data for human cancers

Microarray gene expression data were downloaded for seven cancer types, namely, breast, colon, kidney, lung, pancreatic, prostate and stomach cancer from the GEO database of NCBI [8]. To ensure that our prediction results can be generalized to different datasets, two independent test sets were used to evaluate the robustness of the predicted gene markers obtained from the training set. Detailed information of the data is listed in Table S1. In this study, we have chosen the largest available microarray datasets from each of the seven cancer types, where each dataset includes the (normalized) gene expression levels of each gene in both cancer and control tissues of each patient, along with the stage information for the majority of the cancer samples (some data does not have this information). Note that all the microarray datasets used are normalized using RMA, which has been reported to be more accurately reflective of biological changes compared to other methods like MAS5 (Affymetrix). The distributions of the fold-changes (FC) of individual genes across all genes between cancer and the corresponding control tissues for the seven types of cancers were checked and found to be highly similar. Figure S1 shows one such comparison of FC distributions between breast cancer and lung cancer; hence we believe that comparisons of fold-changes across different cancer datasets in our study are meaningful.

### 2. Identification of differentially expressed genes

For datasets with unpaired cancer and control samples from the same patients, Mann-Whitney test was applied to identify genes that are differentially expressed in cancer *versus* control samples. For those datasets with paired information the test is as follows: Given the hypothesis 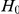 that a particular gene is not differentially expressed in cancer *versus* the control group, the rejection of this hypothesis means that the gene is differentially expressed in cancer. Let 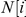 and 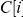, be the gene's expression levels in control and cancer tissues of *i*-th patient, *i = 1… m*, and *m* be the number of patients. It is obvious that if the hypothesis 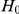 is true, then the probability 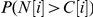 = 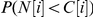 = 0.5, assuming the gene's expression is a continuous random variable. Let *K* be the number of patients with 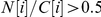, then the random variable *K/m* approximately follows a normal distribution (according to the Central Limit Theorem or de Moivre-Laplace Theorem) with its mean = 0.5 and a standard variation = 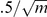, or 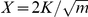 follows a normal distribution *N*(0,1). Thus the *p*-value can be estimated as *P*(*X*>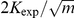), where 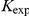 is the number of patients satisfying 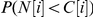. Overall, we consider a gene being differentially expressed if the statistic significance, *p*-value, is less than 0.05 and its fold-change is at least 2.

### 3. Prediction of blood secreted proteins

All genes predicted to be differentially expressed between cancer and the corresponding control samples were analyzed to predict if their proteins are blood-secretory, using a program that our group recently developed [6]. The basic idea of the algorithm is to train a support vector machine (SVM)-based classifier to distinguish between the blood-secretory proteins and proteins that are not secreted, using various sequence-based features such as signal peptides, transmembrane domains, glycosylation sites and polarity measures. On a large independent test set containing 105 secretory proteins and 7,258 non-secretory proteins of humans, the classifier achieved ∼94% prediction sensitivity and ∼98% prediction specificity.

### 4. Prediction of marker genes for each cancer type

For each *k*-gene combination out of the differentially expressed genes defined in the above section, an SVM-based classifier was trained to achieve the highest possible classification accuracy defined as
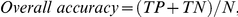
where *TP* and *NP* are the numbers of true positives and negatives, respectively, and *N* is the total number of samples. A linear kernel function was used for training through LIBSVM [9]. For each cancer type, all markers were ranked according to the 5-fold cross-validation performance on the training dataset. In order to find markers that are generalized well to other datasets, we tested the predicted gene markers on two independent test datasets.

### 5. Prediction of markers for multiple cancer types

To identify *k*-gene discriminators for multiple cancer types, all genes that consistently exhibit differential expressions in at least two cancer types were considered. For each *k*-gene combination among these genes, its classification accuracy between each cancer type and the corresponding control tissues was calculated. Then, the *k*-gene combinations exhibiting discerning power across multiple cancer types were determined. The top discriminators for multi-cancer types were selected by using a fixed cut-off on classification accuracies. Throughout the remainder of this paper, *k*-gene groups refer to combinations of *k*-genes for k = 1, 2, 3, 4 unless stated otherwise.

### 6. Pathway enrichment analysis of differentially expressed genes

Functional analysis and pathway enrichment analysis were conducted using DAVID [10], where the pathway information is based on the annotation from KEGG, BBID and BIOCARTA. A *p*-value<0.05 was used to guarantee the significance level of an enriched pathway.

## Results

This study is focused on seven of the most prevalent cancer types in the world, which also have large sets of microarray gene-expression data available in the public domain, collected at a genome scale from tissues of each cancer type as well as from their corresponding noncancerous control tissues. By working on multiple cancer types simultaneously, we can derive potential markers either specific to individual cancer types or general to all or groups of cancers, as well as to identify abnormally activated or deactivated pathways.

### 1. Predicted marker genes for individual cancer types

We have searched for individual genes and gene combinations whose expression patterns can best distinguish between cancer and associated control tissues for each cancer types. Specifically, all 1-, 2-, 3- and 4-gene combinations encoded in the human genome were ranked in terms of their discerning power in distinguishing the cancer samples from the corresponding control samples for each cancer type. In addition, we have also ranked *k*-gene combinations, based on their discerning power between early cancer samples and control samples if the relevant data are available and sufficiently large.

#### A. Breast cancer

The analysis was done on a gene-expression dataset consisting of 43 paired breast cancer and cancer-adjacent control tissues from the same patients [11]. Of the 43 samples, 32 were early-stage cancers (stages I and II). 294 genes were found to be consistently and abnormally expressed with at least a 2-fold change in their expression across the cancer and the control tissues, 81 of which were up-regulated and 213 were down-regulated in the cancer tissues. Among the differentially expressed genes, 69 of their encoded proteins are predicted to be blood secretory by our prediction program [6], and could thus serve as potential serum biomarkers (Supplementary Information File S1).

**Figure 1:**
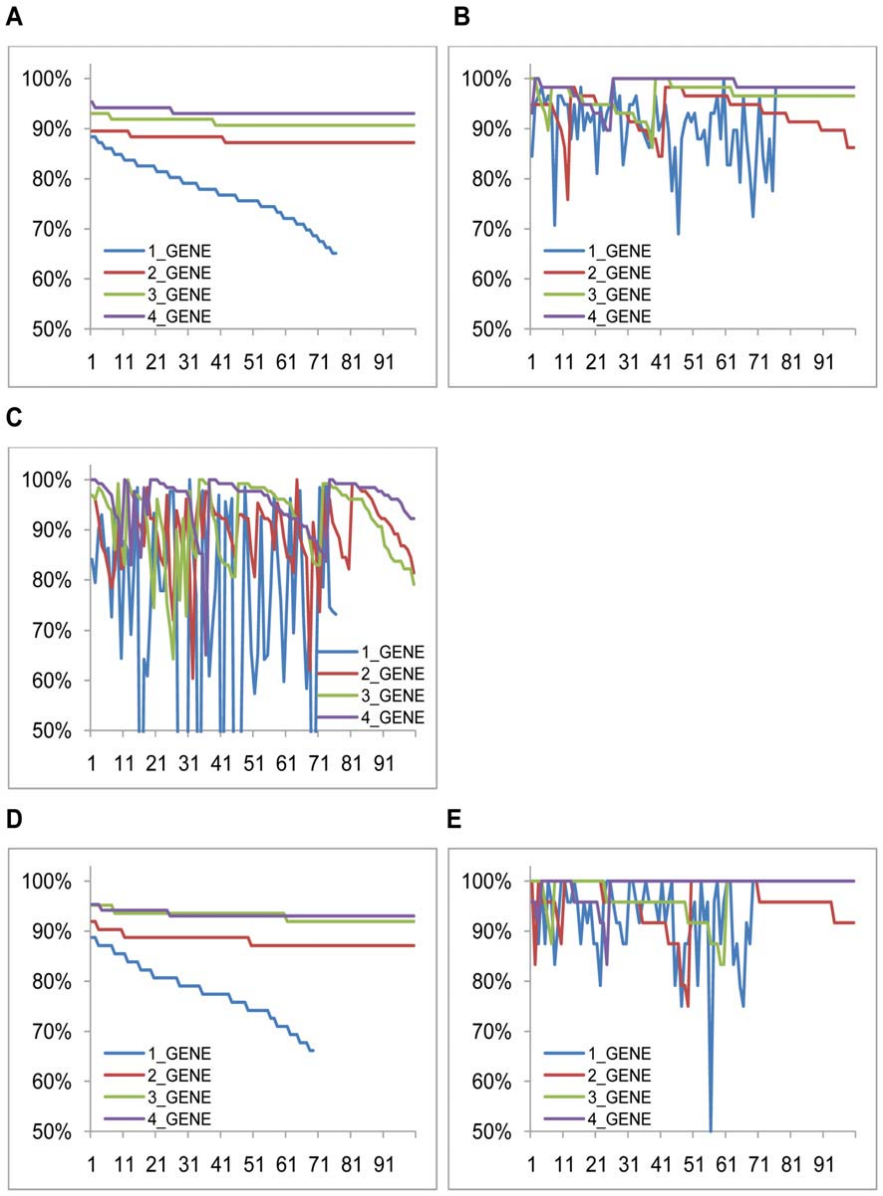
Classification accuracies by the top 100 *k*-gene markers, k = 1, 2, 3, 4, on the training and the test sets of breast cancer. For each panel, the x-axis is the list of 100 *k*-gene markers ordered by their classification performance on the training datasets, and the y-axis represents the classification accuracy. (A) classification accuracies by the top 100 *k*-gene combinations between breast cancer and reference samples in the training set, and (B) and (C) on the two test sets; (D) classification accuracies by top 100 *k*-gene combinations between early breast cancer and corresponding reference samples in the training set and (E) on the test set.

Classification analysis was then conducted (see Materials and Methods), with the goal of identifying *k*-gene combinations whose expression patterns can accurately distinguish between the cancer and the control samples. Figure 1 (A) and (D) show the classification accuracies of the best 100 *k*-gene combinations on the whole training set and on the training set containing only early stage samples, respectively. Two independent evaluation sets are used to assess the generality of the identified gene markers, which consist of 31 and 68 breast cancer, and 27 and 61 control samples [12], respectively. Figure 1 (B) and (C) show the classification performance by the trained classifiers on the two evaluation sets. The detailed list of these 100 *k*-gene combinations is given in Suppplementary Information S1.

As shown in Figure 1, the majority of the top *k*-gene combinations, particularly for *k*>1, perform well on both training and the independent test sets with overall accuracy better than 85% although their ranking orders on the two datasets may not be well preserved. The fluctuations in their classification accuracies are believed to be due to the small size of the training data. Similar observations were made on all the predicted top markers across the seven cancer types.

The best three single gene discriminators are PCOLCE2, ANGPTL4 and LEP, having 88.4%, 88.4% and 87.2% classification accuracy on the training set and 94.8% and 84.1%, 84.5% and 79. 5% and 96.6% and 96.1% on the two test sets, respectively. The top three 2-, 3- and 4-gene combinations are {TACSTD2+CHRDL1, TACSTD2+CAV1, PPARG+TMEM97}, {RRM2+COL1A1+PPARG, RRM2+COL1A1+PCOLCE2, RRM2+GPR109B+SPINT2}, and {RRM2+COL1A1+GPR109B+SPINT2, RRM2+GPR109B+INHBA+SPINT2, TACSTD2+IGFBP6+IGF1+TF}, respectively. Similarly, for early breast cancer, the best three *k*-gene discriminators are {GPR109B, PCOLCE2, PCSK5}, {PCSK5+COL10A1, FERMT2+SPINT2, MAOA+IGJ}, {COL1A1+PCSK5+TF, GPX3+COL1A1+SPINT2, GPX3+FAP+TMEM97}, and {RRM2+COL1A1+GPR109B+IGJ, RRM2+COL1A1+GPR109B+IGJ, RRM2+COL1A1+GPR109B+SPINT2}, respectively.

Although the best three discriminators represent novel discoveries, we noticed some lower-ranked genes have been considered as possible breast cancer markers by previous studies. For example, ADIPOQ (adiponectin) is found to be closely associated with a breast-cancer risk [13]. The SPINT2, an inhibitor of HGF activator, was reported to have higher expression levels in early stage breast cancer and associated with a poor prognosis [14], consistent with our findings. Some others are involved in the activities of cancer cells in general. For example, CAV1, down-regulated in the cancer samples, was found to inhibit breast cancer growth and metastasis [15]; the down-regulation of PPARG is associated with local recurrence and metastasis in breast cancer [16]; and ANGPTL4 may act as a regulator of angiogenesis [17]. To the best our knowledge, all the 2-, 3- and 4-gene discriminators represent novel discoveries.

Similar analyses have been carried out on six other cancer types. The key findings on each of these six cancer types are highlighted below, with the summary being given in Table S2 and gene names listed in Supplementary Information File S1. In addition, Supplementary Information File S2 show the classification accuracies by the best 100 *k*-gene discriminators on both the training and the testing sets for each cancer type, respectively.

#### B. Colon cancer

Our analysis was done on a microarray dataset consisting of 53 colon cancer and 28 cancer-adjacent control tissues from the same patients (some of the cancer samples have no reference samples) [18]. 247 genes were found to be consistently and abnormally expressed with at least a 2-fold change in their expression across the cancer and the control tissues in our training data, 56 of which are up-regulated and 191 are down-regulated in colon cancer tissues. Two independent test sets, consisting of 24 and 22 colon cancer and 24 and 20 cancer-adjacent control samples from the same patients [19], respectively, were used to assess the generality of the predicted markers.

We found the best three single-gene discriminators for colon cancer are MMP7, DPT and MMP1 having 97.5%, 96.3% and 95.1% classification accuracy on the training set, and 97.9% and 90.9%, 97.9% and 74.6%, and 91.7% and 84.1% on the two test sets, respectively. The top three 2-gene discriminators are SLIT3+MMP7, MATN2+MMP7, and MMP7+PTGS1. Some of our top discriminators have been previously studied in the context of colorectal cancer. For example, MMP1 is an invasion-promoting factor, and its up-regulation, as observed in our data, is associated with the invasiveness of the cancer [20]. MMP7 is known to play an important role in cancer growth, and its up-regulation could be a key mechanism for cancer cells' escape from the immune surveillance [21].

#### C. Kidney cancer

The analysis was carried on a microarray gene-expression dataset consisting of 49 kidney cancer and 23 cancer-adjacent control tissue samples from the same patients [22]. 231 genes were found to be consistently and abnormally expressed with at least a 2-fold change in their expression across the cancer and control tissues in our training data, 129 of which are up-regulated and 102 are down-regulated in cancer. Two independent evaluation sets, consisting of 35 and 36 kidney cancer samples and 12 and 9 cancer-adjacent control samples from the same patients, respectively, were used to assess the generality of the predicted markers [23], [24]. The best three single gene discriminators are found to be UMOD, ACPP and CCL18 for kidney cancer, having the same classification accuracy, 98.6% on the training set and 100% and 94.4%, 95.7% and 86.11% and 89.4% and 68.1% on the two test sets, respectively. The top three 2-gene combinations are EGF+ALB, ACPP+UMOD, and UMOD+ALB. Among the top discriminators, UMOD has been reported to be related to kidney disease [25]. SERPINA5, down-regulated in the cancer, regulates the invasive potential of renal cancer growth and invasion. Other top discriminators represent new discoveries. For example, AFM has not been reported to be related to cancer, and C6orf155 does not have a characterized function.

#### D. Lung cancer

The analysis was done on a microarray dataset consisting of 58 lung cancer tissue and 49 cancer-adjacent control tissue samples from the same patients [26]. 683 genes were found to be consistently and abnormally expressed with at least a 2-fold change in their expression across the cancer and control tissues in our training data, 255 of which are up-regulated and 428 are down-regulated in lung cancer tissues. Two independent sets, consisting of 27 and 20 lung cancer and 27 and 19 cancer-adjacent control samples from the same patients [27], was used to assess the generality of the predicted markers.

The best three single gene discriminators are CAV1, SFTPC and VWF for lung cancer, having the same classification accuracy, 99.1% on the training set and 98.2% and 100%, 96.3% and 82.5%, and 88.9% and 100% on the two test sets, respectively. The top three 2-gene combinations are FERMT2+GREM1, TEK+NFASC, CAV1+MMP1. Among the top discriminators, CAV1 has been found to be down-regulated in breast cancer [28], and has been reported to be associated with metastasis in lung cancer [29]. SFTPC has been reported to be associated with interstitial lung disease [30]. FAM107A, which suppresses cell growth, may play a role in cancer development [31]. Other top discriminators represent new observations. For examples, TNXB, SPP1 and EMCN have not previously been reported as cancer-related.

#### E. Pancreatic cancer

The analysis was done on a microarray dataset consisting of 39 paired pancreatic cancer and cancer-adjacent control tissue samples from the same patients [32]. 885 genes were found to be consistently and abnormally expressed with at least a 2-fold change in their expression across the cancer and control tissues in the training data, 616 of which are up-regulated and 269 are down-regulated in pancreatic cancer. Two independent sets, consisting of 36 and 29 pancreatic cancer samples and 16 and 5 cancer-adjacent control samples from the same patients [33], was used to assess the generality of the predicted markers.

The best three single-gene discriminators are KRT17, COL10A1 and CTHRC1 for pancreatic cancer, having the same classification accuracy, 93.6% on the training set and 88.5% and 80.4%, 84.6% and 73.2%, and 84.6% and 85.7% on the two test sets, respectively. The top three 2- and 3-gene discriminators are {MMP7+AZGP1; MMP7+FGL1; MMP7+PLA2G1B} and {CTHRC1+SGPP2+CCL18; TNFRSF21+EGFL6+CTHRC1; COL10A1+S100A6+RSAD2}, respectively. Among the top discriminators, KRT17 is known to be involved in tissue repair [34]. AZGP1 has been reported to cause extensive loss of fat, often associated with advanced cancers [35]. Other top discriminators represent new findings. For examples, RSAD2, involved in antiviral defense, has not been reported as being related to cancer, as well as SGPP2, known to be involved in pro-inflammatory signaling [36], and CST4.

#### F. Prostate cancer

The analysis was done on a microarray dataset consisting of 65 prostate cancer and 63 cancer-adjacent control tissue samples from the same patients [37]. 118 genes were found to be consistently and abnormally expressed with at least a 2-fold change in their expression across the cancer and control tissues in our training data, of which 23 are up-regulated and 95 are down-regulated in lung cancer tissues. Two independent sets, consisting of 62 and 53 prostate cancer samples and 47 and 14 cancer-adjacent control samples from the same patients [38], was used to assess the generality of the predicted markers.

The best three single gene discriminators are MYLK, PALLD and CAV1 for prostate cancer, having 73.4%, 71.9% and 71.1% classification accuracy on the training set and 83.5% and 62.3%, 69.6% and 72.6%, and 94.2% and 75.5% on the two test sets, respectively. The top three 2- and 3-gene discriminators are {LTF+IGF1; LTF+SPARCL1; SMTN+CCK}, {SMTN+CCK+CCL2; SMTN+CCK+COMP; SMTN+CCK+PLA2G7}, respectively. Among the top discriminators, LTF is known to inhibit the growth of tumors [39]. IGF1, a growth factor, plays a role in the development of prostate cancer [40] and has been reported as an indicator of advanced prostate cancer [41]. Other top discriminators represent new discoveries. For example, CHRDL1 may play a role in regulating angiogenesis [42] but has not been reported to be related to cancer. The same is with SMTN.

#### G. Stomach cancer

The analysis was done on a microarray dataset consisting of 89 stomach cancer and 23 cancer-adjacent control tissues from the same patients [43]. Out of the 89 cancer tissue samples, 31 are early-stage cancers. 311 genes were found to be consistently and abnormally expressed with at least a 2-fold change in their expression across the cancer and control tissues in our training data, 166 of which are up-regulated and 145 are down-regulated in lung cancer tissues. Two independent sets, consisting of 38 and 16 stomach cancer samples and 31 and 13 cancer-adjacent control samples from the same patients [44], [45] was used to assess the generality of the predicted markers, of which 12 are early stage samples partially paired with 10 control samples.

The best three single-gene discriminators are SERPINH1, BGN and COL12A1 for stomach cancer, having 99.1%, 98.2% and 98.2% classification accuracy on the training set and 94.2% and 96.7%, 88.4% and 93.3%, and 84.1% and 75.8% on the two test sets, respectively. The top three 2-gene combinations are CHGA+SERPINH1, TGFBI+CHGA and PGC+SERPINH1, respectively. For early stomach cancer, the best three *1*-gene discriminators are also SERPINH1, BGN and COL12A1, respectively. Among the top discriminators, BGN is known to have a role in controlling cell growth in cancer [46]. The abnormal expression of CTHRC1, a regulator of matrix deposition, has been widely found across different solid cancers and is considered to be associated with cancer invasion and metastasis [34]. Of particular interest is that PGC has been proposed as an indicator of gastric cancer [47], and the serum level of PGC was used as a biomarker for precancerous lesions of the stomach [48]. Other top discriminators represent new discoveries. For example, ABCA5, ADAMTS12 and CLEC3B have not been reported to be cancer related.

**Figure 2:**
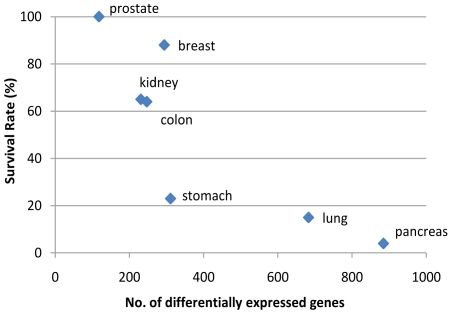
Correlation between 5-year survival rate and the number of differentially genes in each cancer type.

Interestingly, the number of differentially expressed genes across different cancer types has a wide spread, ranging from 118 (prostate), 231 (kidney), 247 (colon), 294 (breast), 311 (stomach) to 683 (lung) and 885 (pancreatic). One possible explanation is that these numbers may reflect the aggressiveness of the corresponding cancers. We did notice that there is strong correlation between the number of differentially expressed genes in a given cancer type and the five-year survival rate of patients with that cancer [49] (Figure 2). The detailed statistics is given in Table S3. Another interesting observation is that, while the majority of the differentially expressed genes with at least a 2-fold change in five cancer types (breast, colon, lung, prostate, stomach) are down-regulated, in kidney and pancreatic cancers, the majority of such genes are up-regulated, possibly suggesting unique characteristics of these two cancer types.

### 2. Markers for multiple cancer types

We have also sought to identify genes that could be used as indicators for cancer in general or for a group of cancers. It is possible to find common gene “markers” across different cancer types because of the observation that the majority of the cancers, if not all, undergo a common set of alterations [50] during oncogenesis, such as self-sufficiency in growth signals, insensitivity to antigrowth signals, evasion of apoptosis, and tissue invasion and metastasis. Some of these biological processes may be executed by the same groups of proteins during the formation and progression of different cancers, hence possibly giving rise to common markers for different cancer types.

#### A. Identification of genes differentially expressed across multiple cancer types

We have examined differentially expressed genes with at least 2-fold changes between cancer and corresponding control tissues across all seven cancer types and attempted to find those genes common to multiple cancer types. The key findings are summarized in Table 1.

**Table 1:** A list of 19 genes that are differentially expressed in more than 4 cancer types and their relevance to different cancer types.

Gene ID	Direction of regulation							Reported to be related to cancers							
	Breast	Colon	Kidney	Lung	Pancreas	Prostate	Stomach	B.	C.	K.	L.	Pa.	Pr.	S.	Other cancer types
CDC2	**↑**	**↑**		**↑**	**↑**		**↑**	*	*		*		*	*	liver cancer; squamous cell carcinoma;nasopharyngeal carcinoma
AURKA	**↑**	**↑**		**↑**		**↑**	**↑**	*	*		*	*	*	*	ovarian cancer;esophageal squamous cancer;uterine cancer;bladder cancer
ABCA8	**↓**	**↓**	**↓**	**↓**			**↓**								
DPT	**↓**	**↓**		**↓**		**↓**	**↓**								
TOP2A	**↑**	**↑**		**↑**	**↑**		**↑**	*	*					*	bladder cancer;ovarian cancer; squamous cell carcinoma
MMP7		**↑**		**↑**	**↑**		**↑**	*	*		*	*		*	ovarian cancer; oral cancer; rectal cancers; bladder cancer; liver cancer
MAD2L1		**↑**		**↑**	**↑**		**↑**		*					*	thyroid carcinomas; oesophageal squamous cancer
KLF4	**↓**	**↓**		**↓**			**↓**	*	*					*	esophageal cancer;bladder cancer
MELK	**↑**			**↑**	**↑**		**↑**	*							brain cancer;endometrial cancer
C7		**↓**	**↓**	**↓**		**↓**		*					*		uterine cervical cancers
ECT2		**↑**		**↑**	**↑**		**↑**					*			
PRC1	**↑**			**↑**	**↑**		**↑**	*							
RRM2	**↑**			**↑**	**↑**		**↑**				*	*	*		
ALDH1A1	**↓**	**↓**		**↓**			**↓**					*			non-small cell bronchopulmonary cancer; liver cancer; T-cell leukemia
PMAIP1	**↑**	**↑**		**↑**	**↑**				*		*	*			
FABP4	**↓**	**↓**		**↓**			**↓**	*							Bladder cancer;
COL11A1	**↑**	**↑**		**↑**	**↑**										adenomas;
TTK		**↑**		**↑**		**↑**	**↑**								
CENPF	**↑**			**↑**	**↑**		**↑**	*							

“↑” indicates up-regulated gene expression in the corresponding cancer type while “↓”is down-regulation. “*” indicates that a gene has been reported as relevant to the corresponding cancer type. “B.” for breast cancer; “C.” for colon cancer; “K.” for kidney cancer”; “L.” for lung cancer”; “Pa.” for pancreatic cancer”; “Pr.” for prostate cancer” and “S.” for stomach cancer”.

85 genes are found to be differentially expressed across at least three cancer types (Table S4), among which 19 genes are across at least four cancer types, and five genes (ABCA8, DPT, FHL,CDC2 and TOP2A) across five cancer types. The differences in the gene expression across different cancer types may indicate either a general or specific relevance of the gene to the corresponding cancers, which has been partially confirmed by the functional analysis and an extensive literature search. The detailed molecular function of these genes is summarized in Table S4. 63 out of the 85 genes have been reported to be cancer associated by previous studies. For example, CDC2, up-regulated in five of the seven cancers studied, has been reported to be related to colon, prostate and stomach cancer, which is not surprising in view of its role in regulating the cell cycle, e.g. entry from G_1_ to S; TOP2A, again up-regulated in five of the seven cancers, has been reported to be associated with gastric [51], breast [52] and ovarian cancer [53], consistent with its function in DNA strand regulation; Both of these two genes have been considered as multi-type cancer markers by a previous meta-analysis of cancer microarray data [7]. RRM2, up-regulated in four of the seven cancers, has been suggested to be related to esophageal and gastric cancers and prostate cancer, consistent with its critical role in DNA synthesis which must be maintained in rapidly dividing cells. In addition, 49 genes have been reported to be relevant to immune diseases, such as CXCL12, COL1A1, MMP9, and CD36 [54], [55], [56], [57], likely reflecting an inflammatory–type response often associated with cancer. Among them, MMP9, important in extracellular matrix degradation, is up-regulated in three of the seven cancers, and CD36, which may function in cell adhesion, is down-regulated in three of the seven cancers; both of these changes are consistent with a role of the gene products in metastasis.

#### B. Pathway enrichment analysis of differentially expressed genes

We have carried out a pathway enrichment analysis on genes that are differentially expressed in any of the seven cancer types. Overall, a number of signaling pathways are consistently and highly enriched across all seven types of cancers, such as Wnt, p53 and integrin signaling pathways, as well as a few other processes like phospho-APC/C-mediated degradation of cyclin A and inflammation determined by chemokine and cytokine signaling pathways (in addition to the general cellular processes such as cell cycle, DNA replication and repair, apoptosis and various metabolic pathways). Notably, these pathways are mostly enriched with up-regulated genes in cancer, suggesting a possible activation of these processes. In addition, a few metabolic pathways such as tyrosine, histidine, phenylalanine, butanoate and 5-hydroxytryptamine pathways are enriched only with down-regulated genes across all cancers. This may indicate a possible deficiency of the relevant metabolic enzymes in cancer, which could, for example, arise from loss-of-function mutations in their genes. These observations may suggest the essential roles played by these processes in cancer formation and progression.

Other than the above processes common to all cancers, a few pathways are enriched only in specific cancers. For example, arginine, proline, glutamate and riboflavin (vitamin B2) metabolisms are enriched with up-regulated genes only in lung cancer; folate biosynthesis and nitrogen metabolism pathways are enriched in breast cancer; formyltetrahydroformate biosynthesis in stomach cancer; and NF-kB activation and Csk activation by cAMP-dependent protein kinase inhibits signaling through T-cell receptor in kidney cancer. Of particular interest is the finding that pancreatic cancer has the greatest number of differentially expressed genes, compared to other cancer types, that are involved in a complex network consisting of the EGF signaling pathway, purine and aminosugar metabolism, PKC-catalyzed phosphorylation of inhibitory phosphoprotein of myosin phosphatase, metabotropic glutamate receptor group II pathway, Fc epsilon receptor I signaling and the BCR and IL 4 signaling pathways. This suggests a highly active state of the underlying cells in terms of cell growth, differentiation, invasion and metastasis, consistent with the known aggressiveness of the cancer. Seeking the genes and their products that are responsible for the more aggressive behaviors of pancreatic cancer may provide new targets for treating the cancer or preventing the cancer from progression.

A number of pathways specific to a group of cancers have also been identified, which may suggest common characteristics of the underlying neoplasms. For example, the glutathione metabolic pathway is enriched across five cancer types, excluding breast and prostate cancer; *E. coli* infection-related pathways are activated in kidney, lung, pancreatic and stomach cancers but not in other cancers; the thyrotropin-releasing hormone receptor signaling pathway is activated in pancreatic and kidney cancer, but not in the other five cancers; and steroid biosynthesis is activated in breast, lung and pancreatic cancer but not in the other four cancers. Cancer-specific pathway activations have been previously reported. For example, the thyrotropin-releasing hormone receptor signaling pathway was reported to promote programmed cell death in pancreatic cancer [58]; steroid biosynthesis in pancreatic cancer was found based on analyses of several steroidogenic enzymes, such as the cytochrome P-450scc enzymatic complex (P450scc) that is responsible for the conversion of cholesterol into pregnenolone.

These diverse findings indicate that comparative analyses of cancer microarray data can reveal interesting and undetected relationships across different cancer types/subtypes, thus providing useful guiding information for further investigation. The detailed pathway-enrichment information across different cancer types is summarized in Table S5.

#### C. Top k-gene markers for multiple cancer types

We have examined the *k*-gene combinations among genes that are differentially expressed in each cancer type to find gene combinations that are common to multiple cancer types. The idea is to identify commonalities of gene combinations with differential expression patterns between cancer and corresponding control tissue across multiple cancer types, which could provide useful information about common underlying mechanisms of carcinogenesis of different cancers.

Table 2 gives the top two 2-gene combinations across at least three cancer types. CDC2+DPT and CDC2+TOP2A are found to be good discriminators for five cancer types, namely breast, colon, lung, prostate and stomach cancers. Similarly, ABCA8+ALDH1A1+DPT and ABCA8+AURKA+DPT are good 3-gene discriminators for four cancer types with higher classification accuracies than the top 2-gene discriminators, as shown in Table S6.

**Table 2:** The top 2-gene markers for multiple cancer types with each numerical value showing the classification accuracy between a cancer and its corresponding control tissue.

Count	Markers	Breast			Colon			Kidney			Lung			Pancreas			Prostate			Stomach		
		train	test	test2	train	test	test2	train	test	test2	train	test	test2	train	test	test2	train	test	test2	train	test	test2
5	CDC2+TOP2A	72.4	94.8	95.3	75.0	100	64.3	_	_	_	85.2	85.2	79.5	71.2	71.2	87.5	_	_	_	78.3	85.5	85.2
4	CDC2+DPT	70.7	94.8	96.1	91.7	97.9	64.3	_	_	_	88.9	92.6	82.1	_	_	_	_	_	_	66.7	85.5	85.2
	CDC2+ECT2	_	_	_	85.4	97.9	69.0	_	_	_	83.3	77.8	miss	78.8	86.5	87.5	_	_	_	75.4	78.3	65.6
	ABCA8+AURKA	81.0	96.6	99.2	91.7	100	N.A.	_	_	_	94.4	94.4	92.3	_	_	_	_	_	_	75.4	92.8	74.1
	ABCA8+FABP4	79.3	96.6	91.5	89.6	97.9	85.7	_	_	_	96.3	98.1	94.9	_	_	_	_	_	_	79.7	84.1	66.7
	DPT+FABP4	79.3	87.9	85.2	95.8	89.6	65.2	_	_	_	94.4	96.3	94.9	_	_	_	_	_	_	82.6	75.4	81.5
	FABP4+TOP2A	77.6	94.8	93.0	85.4	100	67.6	_	_	_	96.3	94.4	92.3	_	_	_	_	_	_	78.3	85.5	88.9
3	CDC2+SULF1	_	_	_	_	_	_	_	_	_	90.7	88.9	76.9	96.2	90.4	87.5	_	_	_	95.7	88.4	77.8

Each entry represents the classification accuracy (by percentage) between a cancer set and its corresponding reference set on the training (train) and the testing (test) datasets, respectively. (N.A. : the platform of the extra test data doesn't cover the corresponding gene).

As noted, CDC has been reported to play a key role in cell proliferation and apoptosis [59], and DPT is suggested to have a possible role in carcinogenesis through its interaction with a known oncogene, TGFB1. Moreover, some of the top discriminator genes have been reported to be cancer relevant. For example, ECT2 is reported to be involved in cancer development, influencing processes such as the cell cycle, apoptosis and cell division [60]; FABP4 is involved in the activation of the immune response and is reported to be related to breast cancer [61] and bladder cancer [62]; and TOP2A is involved in stomach cancer [51]. These independent observations confirm that the findings herein are meaningful.

#### D. Top k-gene markers that are blood secretory

By combining our blood-secretion prediction capability [6] with the above top gene discriminators, we have predicted proteins that may be secreted into circulation, thus providing candidate serum marker proteins for cancer detection. Table 3 summarizes the top *k*-gene discriminators that are predicted to have their proteins secreted into blood. Some genes involved in these top candidate discriminators have been previously reported to be cancer related, e.g. MMP7 [63]. Other predicted blood-secretory marker proteins such as PAICS, CHRDL1, KLF2, COL10A1 and MYL9 have not heretofore been reported to be cancer related.

**Table 3:** Top *k*-gene discriminators with their proteins being blood secretory.

	Markers	Breast			Colon			Kidney			Lung			Pancreas			Prostate			Stomach		
		train	test	test2	train	test	test2	train	test	test2	train	test	test2	train	test	test2	train	test	test2	train	test	test2
3	GREM1+MMP7	_	_	_	_	_	_	_	_	_	88.9	79.6	64.1	92.3	73.1	87.5	_	_	_	89.9	75.4	63.0
3	MMP7+MMP9	_	_	_	_	_	_	_	_	_	75.9	79.6	87.2	96.2	78.9	87.5	_	_	_	77.9	76.8	70.4
3	MMP11+MMP7+MMP9+RRM2	_	_	_	_	_	_	_	_	_	85.2	96.3	87.2	96.2	88.5	87.5	_	_	_	88.1	88.4	84.4
3	CCL18+TGFBI	_	_	_	_	_	_	74.5	80.9	80.0	_	_	_	82.7	82.7	87.5	_	_	_	71.0	75.4	77.8
3	DPT+MMP7	_	_	_	97.9	89.6	76.2	_	_	_	85.2	88.9	87.2	_	_	_	_	_	_	84.2	81.2	74.1
3	FAM107A+KLF4	_	_	_	87.5	100	miss	_	_	_	94.4	92.6	94.9	_	_	_	_	_	_	91.3	92.8	70.4
3	FAM107A+KLF4+MMP7+PAICS	_	_	_	100	100		_	_	_	94.4	94.4	94.9	_	_	_	_	_	_	91.3	91.3	84.4
3	INHBA+RRM2	74.1	100	98.4	_	_	_	_	_	_	_	_	_	94.2	88.5	87.5	_	_	_	78.3	81.2	74.1
3	GPX3+RRM2	81.0	96.6	96.1	_	_	_	_	_	_	88.9	94.4	94.9	_	_	_	_	_	_	85.5	81.2	77.8
3	COL11A1+DPT	72.4	96.6	96.9	97.9	89.6	57.1	_	_	_	92.6	94.4	87.2	_	_	_	_	_	_	_	_	_
3	MMP11+RRM2	_	_	_	_	_	_	_	_	_	88.9	90.7	69.2	86.5	88.5	87.5	_	_	_	75.0	82.6	63.0

Each numerical value represents the classification accuracy (by percentage) between cancer tissues and their corresponding reference tissues.

While Table 3 gives a detailed list of all the gene combinations whose proteins are predicted to be blood secretory, with discerning power between cancer and corresponding reference tissues higher than 70%, a few top candidates for these seven cancer types are highlighted. Three types of cancers are covered by 22 2-gene combinations, with MMP11+RRM2 and MMP7+MMP9 representing the top 2-gene markers with at least 75% classification accuracy. The best 4-gene combination, MMP7+MMP9+MMP11+RRM2, gives at least 86% classification accuracy for lung, pancreatic and stomach cancers, and all of these four genes are up-regulated by at least 2-fold in the cancer tissues, suggesting the potential of this combination as a good blood marker for these cancer types. CCL18+TGFBI represents a good discriminator for kidney, pancreatic and stomach cancer, which are up-regulated by at least 2-fold in cancer tissues. Similarly, CN2+THBS2 are both up-regulated by 2-fold in kidney, lung and pancreatic cancer. MMP11+RRM2 are up-regulated in lung cancer, pancreatic cancer and stomach cancer tissues, and hence may also make a good marker for these three cancer types.

### Concluding Remarks

A computational protocol for predicting gene markers in cancer tissues and protein markers in sera was developed for simultaneous analyses of multiple cancer types. In addition to individual gene markers, we have focused on gene combinations that can be used to distinguish multiple cancer types from their corresponding control tissues. The pathway enrichment analysis among the differentially expressed genes across multiple cancer types, as well as those specific to individual cancer types, has identified a number of abnormally activated or deactivated pathways across multiple cancers and for specific cancers. The information provided on individual genes and pathways, along with potential serum biomarkers, should provide highly useful information for elucidating pathways in cancer, as well as expediting the search for potential serum biomarkers of specific cancers.

## Supporting Information

File S1 Links to the supplementary tables for the top k-gene markers for 7 individual cancer types.
(0.03 MB DOC)

File S2 The Supplementary Figures for the top k-gene markers for 7 individual cancer types
(0.54 MB DOC)

Table S1 A summary of the training and the testing set used in our analysis
(0.03 MB DOC)

Table S2 A summary of the top three k-gene discriminators, k = 1, 2, 3, 4, for each of the seven cancer types along with discriminators for early stage breast and stomach cancer
(0.04 MB DOC)

Table S3 Statistics of 5-year relative survival rates by race and year of diagnosis, US. 1974–2001
(0.04 MB DOC)

Table S4 The list of 85 genes that differentially expressed in more than 3 cancer type
(0.19 MB DOC)

Table S5 Enriched pathways by differentially expressed genes in different cancer types (enrichment P-value cutoff = 0.05)
(0.09 MB DOC)

Table S6 The top 3-gene and 4-gene discriminators for multiple cancer type types
(0.06 MB DOC)

Figure S1 Comparison of the gene expression fold changes (A) between breast cancer training and testing datasets, and (B) between breast cancer and lung cancer
(0.30 MB TIF)
