# Supplementary material for: A Comparative Analysis of Gene-Expression Data of Multiple Cancer Types
Source: PLoS One. 2010 Oct 27;5(10):e13696. doi: 10.1371/journal.pone.0013696 (PMC2965162; doi:10.1371/journal.pone.0013696)
Supplement: File S1 — Links to the supplementary tables for the top k-gene markers for 7 individual cancer types. (0.03 MB DOC) [file pone.0013696.s001.doc]

**Supporting information S1**

**Table A1.** The detailed list of 100 *k*-gene 100 combinations for **Figure 1** (a)(b) breast cancer

<http://csbl.bmb.uga.edu/publications/materials/kunxu/PAPER_1/BREAST_CANCER/breast_tn_blood_1to4.htm>

**Table A2.** The detailed list of 100 *k*-gene combinations for **Figure 1** (c) (d) early stage breast cancer

<http://csbl.bmb.uga.edu/publications/materials/kunxu/PAPER_1/BREAST_CANCER/BREAST_STAGE_1_TO_4.htm>

**Table B.** The detailed list of 100 *k*-gene combinations for **Figure 2** (a)(b) colon cancer

<http://csbl.bmb.uga.edu/publications/materials/kunxu/PAPER_1/COLON_CANCER/colon_1to4_marker.htm>

**Table C.** The detailed list of 100 *k*-gene combinations for **Figure 3** (a)(b) kidney cancer

<http://csbl.bmb.uga.edu/publications/materials/kunxu/PAPER_1/KIDNEY_CANCER/kidney_1to4_chart.htm>

**Table D.** The detailed list of 100 *k*-gene combinations for **Figure 4** (a)(b) lung cancer

<http://csbl.bmb.uga.edu/publications/materials/kunxu/PAPER_1/LUNG_CANCER/LUNG_CANCER_1to4.htm>

**Table E.** The detailed list of 100 *k*-gene combinations for **Figure 5** (a)(b) pancreatic cancer

<http://csbl.bmb.uga.edu/publications/materials/kunxu/PAPER_1/PANCREATIC_CANCER/PANCREASE_CANCER_1TO4xlsx.htm>

**Table F.** The detailed list of 100 *k*-gene combinations for **Figure 6** (a)(b) prostate cancer

<http://csbl.bmb.uga.edu/publications/materials/kunxu/PAPER_1/PROSTATE_CANCER/PROSTATE_1to4_MARKER_CHART.htm>

**Table G1.** The detailed list of 100 *k*-gene combinations for **Figure 7** (a)(b) stomach cancer

<http://csbl.bmb.uga.edu/publications/materials/kunxu/PAPER_1/STOMACH_CANCER/STOMACH_1_to_4_CHART.htm>

**Table G2.** The detailed list of 100 *k*-gene combinations for **Figure 7** (c)(d) early stage stomach cancer

<http://csbl.bmb.uga.edu/publications/materials/kunxu/PAPER_1/STOMACH_CANCER/stamach_stage_1to4.htm>
