# Supplementary material for: A Comparative Analysis of Gene-Expression Data of Multiple Cancer Types
Source: PLoS One. 2010 Oct 27;5(10):e13696. doi: 10.1371/journal.pone.0013696 (PMC2965162; doi:10.1371/journal.pone.0013696)

**Supporting Information S2**

**Figure 1**: classification performance by top 100 k-gene groups, k = 1, 2, on the training and test sets of colon cancer. (A) classification accuracies by the top 100 *k*-gene combinations between colon cancer and reference samples in the training set. (B) and (C) classification accuracies by the top 100 *k*-gene combinations on the two test sets.

**A B C**

**
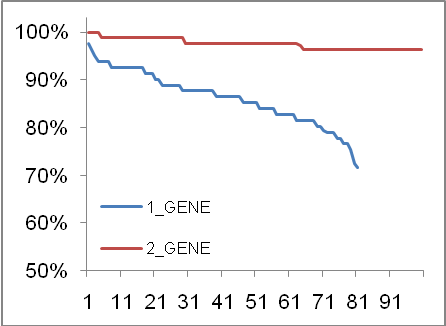

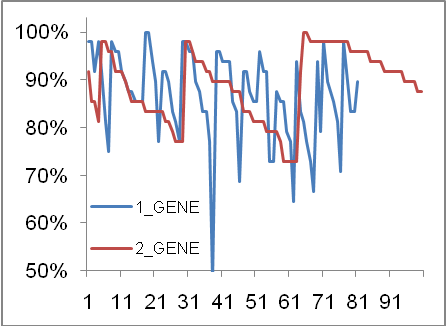

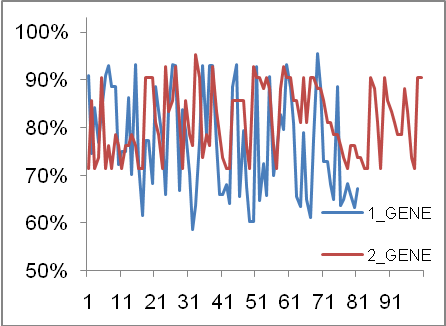
**

**Figure 2**: classification performance by top 100 k-gene groups, k = 1, 2, on the training and test sets of kidney cancer. (A) classification accuracies by the top 100 *k*-gene combinations between kidney cancer and reference samples in the training set. (B) and (C) classification accuracies by the top 100 *k*-gene combinations on two test sets.

**A B C**

**
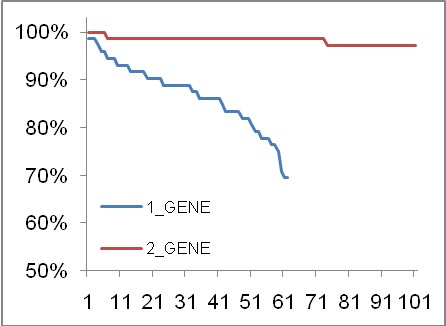

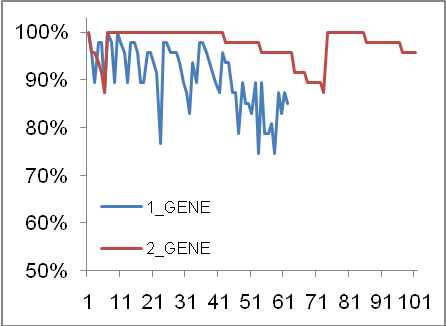

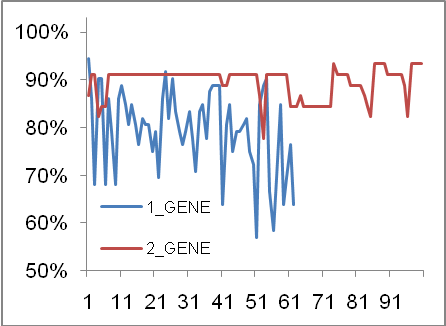
**

**Figure 3**: classification performance by top 100 k-gene groups, k = 1, 2, on the training and test sets of lung cancer. (A) classification accuracies by the top 100 *k*-gene combinations between lung cancer and reference samples in the training set. (B) and (C) classification accuracies by the top 100 *k*-gene combinations on the two test sets.

**A B C**

**
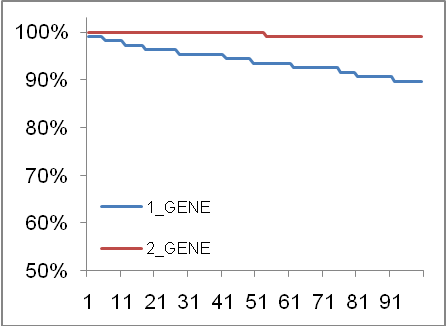

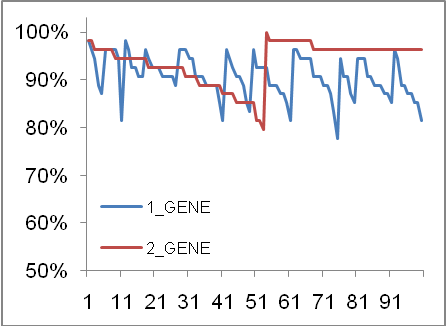

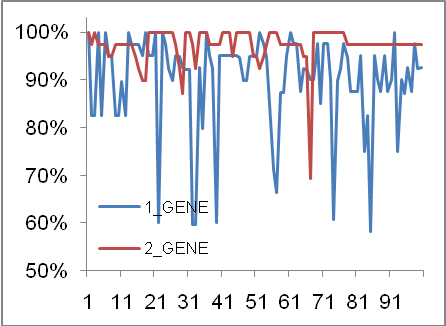
**

**Figure 4**: classification performance by top 100 k-gene groups, k = 1, 2, on the training and test sets of pancreatic cancer. (a) classification accuracies by the top 100 *k*-gene combinations between pancreatic cancer and reference samples in the training set. (b) and (c) classification accuracies by the top 100 *k*-gene combinations on the two test sets.

**A B C**

**
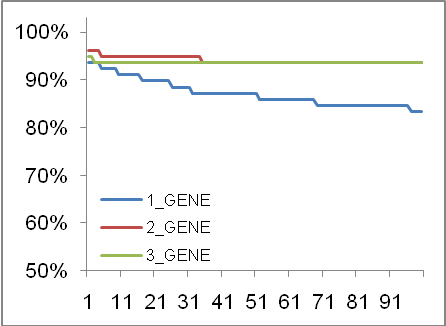

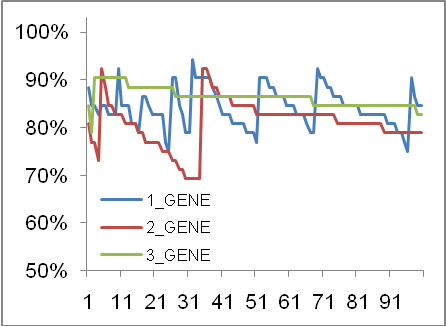
**
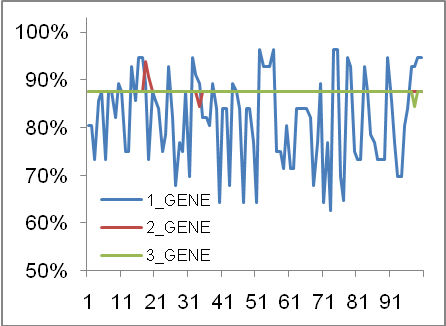


**Figure 5**: classification performance by top 100 k-gene groups, k = 1, 2, on the training and test sets of prostate cancer. (a) classification accuracies by the top 100 *k*-gene combinations between prostate cancer and reference samples in the training set. (b) and (c) classification accuracies by the top 100 *k*-gene combinations on the two test sets.

**A B C**

**
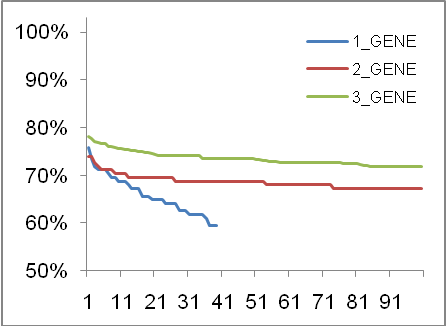

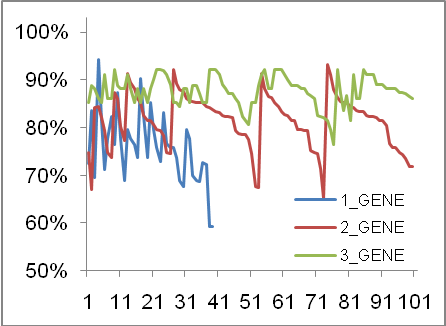
**
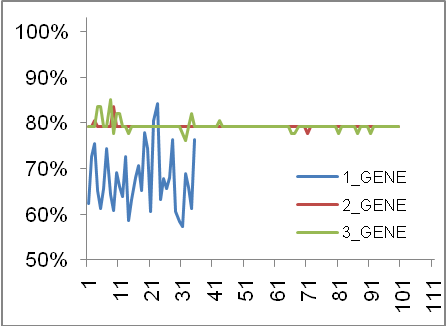


**Figure 6.** Classification accuracies by the top 100 *k*-gene markers, k = 1, 2, 3, 4, on the training and the test sets of stomach cancer. . (a) classification accuracies by the top 100 *k*-gene combinations between stomach cancer and reference samples in the training set. (b) and (c) classification accuracies by the top 100 *k*-gene combinations on the two test sets. (d) classification accuracies by top 100 *k*-gene combinations between early stomach cancer and corresponding reference samples in the training set and (e) on the test set.

**A B C**


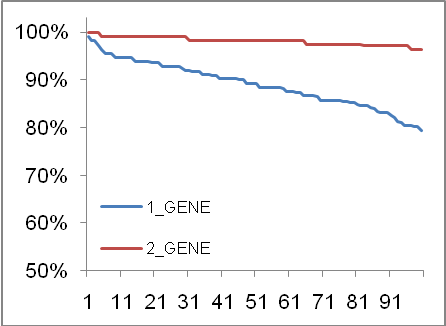

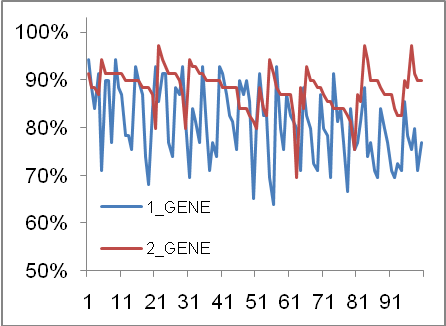

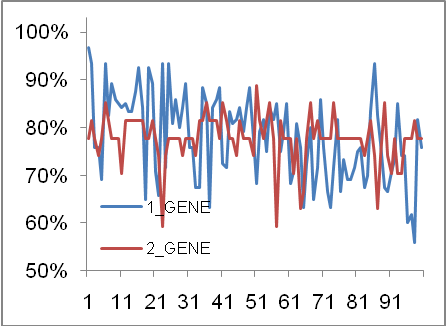


**D E**


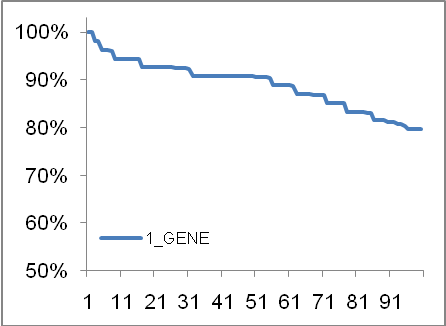

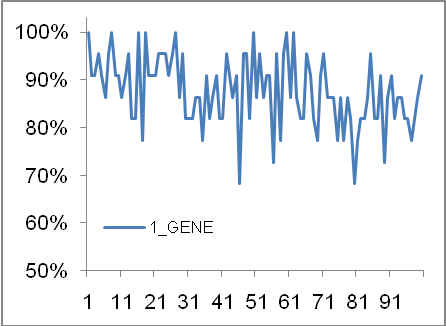

Supplement: File S2 — The Supplementary Figures for the top k-gene markers for 7 individual cancer types (0.54 MB DOC) [file pone.0013696.s002.doc]
