# Supplementary material for: A Comparative Analysis of Gene-Expression Data of Multiple Cancer Types
Source: PLoS One. 2010 Oct 27;5(10):e13696. doi: 10.1371/journal.pone.0013696 (PMC2965162; doi:10.1371/journal.pone.0013696)
Supplement: Table S1 — A summary of the training and the testing set used in our analysis (0.03 MB DOC) [file pone.0013696.s003.doc]

**Table S1:** A summary of the training and the testing set used in our analysis

| Cancer | GEO dataset ID  training / testing data /extra testing data | # reference/ #cancer samples |
| --- | --- | --- |
| breast cancer | GSE15852 / GSE10810 / GSE14999 | 43/43 (27/31) (61/68) |
| colon cancer | GSE6988 / GSE10950 / GSE15781 | 28/53 (24/24) (20/22) |
| kidney cancer | GSE15641 / GSE4866 / GSE17816 | 23/49 (12/35) (9/36) |
| lung cancer | GSE10072 / GSE7670 / GSE2514 | 49/58 (27/27) (19/20) |
| pancreatic cancer | GSE15471 / GSE16515 / GSE11838 | 39/39 (16/36) (5/29) |
| prostate cancer | GSE6606 / GSE3933 / GSE14206 | 63/65 (47/62) (14/53) |
| stomach cancer | GSE2701 / GSE13911 / GSE19826 | 23/89 (31/38) (13/16) |
