# Supplementary material for: A Comparative Analysis of Gene-Expression Data of Multiple Cancer Types
Source: PLoS One. 2010 Oct 27;5(10):e13696. doi: 10.1371/journal.pone.0013696 (PMC2965162; doi:10.1371/journal.pone.0013696)
Supplement: Table S2 — A summary of the top three k-gene discriminators, k = 1, 2, 3, 4, for each of the seven cancer types along with discriminators for early stage breast and stomach cancer (0.04 MB DOC) [file pone.0013696.s004.doc]

**Table S2:** A summary of the top three *k*-gene discriminators, k = 1, 2, 3, 4, for each of the seven cancer types along with discriminators for early stage breast and stomach cancer

| Cancer type | No. of samples (C/R) in  training and testing set | Top three k-gene discriminators | | | |
| --- | --- | --- | --- | --- | --- |
|  |  | 1-gene | 2-gene | 3-gene | 4-gene |
| Breast cancer | 43/43  31/27  68/61 | PCOLCE2;  ANGPTL4;  LEP; | TACSTD2+CHRDL1;  TACSTD2+CAV1;  PPARG+TMEM97 | RRM2+COL1A1+PPARG;  RRM2+COL1A1+PCOLCE2;  RRM2+GPR109B+SPINT2; | RRM2+COL1A1+GPR109B+SPINT2;  RRM2+GPR109B+INHBA+SPINT2, TACSTD2+IGFBP6+IGF1+TF |
| Breast cancer  Early stage | 31/31  12/12 | GPR109B ;PCOLCE2 ;  ADIPOQ; | PCSK5+COL10A1, FERMT2+SPINT2, MAOA+IGJ | COL1A1+PCSK5+TF, GPX3+COL1A1+SPINT2, STBD1+TMEM97+COL10A1 | RRM2+COL1A1+GPR109B+IGJ, RRM2+ COL1A1+ GPR109B+SPINT2  COL1A1+MAOA+SPINT2+COL11A1 |
| Colon cancer | 53/28  24/24  22/20 | MMP7;  DPT;  MMP1; | SLIT3+MMP7  MATN2+MMP7;  MMP7+PTGS1; | --- | --- |
| Kidney cancer | 49/23  35/12  36/9 | UMOD; CCL18;  ACPP; | EGF+ALB;  ACPP+UMOD;  UMOD+ALB; | --- | --- |
| Lung cancer | 58/49  27/27  20/19 | CAV1; SFTPC;  VWF; | FERMT2+GREM1 ;  TEK+NFASC ;  CAV1+MMP1 ; | --- | --- |
| Pancreatic cancer | 39/39  36/16  29/5 | KRT17; COL10A1; CTHRC1; | MMP7+AZGP1; MMP7+ELA3B; MMP7+PLA2G1B; | CTHRC1+SGPP2+CCL18;  TNFRSF21+EGFL6+CTHRC;  COL10A1+S100A6+RSAD2; | --- |
| Prostate  cancer | 65/63  62/47  53/14 | MYLK ;  PALLD ;  CAV1; | LTF+IGF1 ;  LTF+SPARCL1 ;  SMTN+CCK ; | SMTN+CCK+CCL2 ; SMTN+CCK+COMP ; SMTN+CCK+PLA2G7 ; | --- |
| Stomach  cancer | 89/23  38/31  13/16 | SERPINH; BGN;  COL12A1; | CHGA+SERPINH1; TGFBI+CHGA;  PGC+SERPINH1; | --- | --- |
| Stomach cancer  Early stage | 31/23  12/10 | SERPINH;  BGN;  COL12A1; | --- | --- | --- |

“C” for cancer and “R” for reference tissues; and “---“ indicates that the corresponding *k*-gene combinations were not assessed since (*k-1*)-gene combinations already give 100 classification accuracy.
