# Supplementary material for: A Comparative Analysis of Gene-Expression Data of Multiple Cancer Types
Source: PLoS One. 2010 Oct 27;5(10):e13696. doi: 10.1371/journal.pone.0013696 (PMC2965162; doi:10.1371/journal.pone.0013696)
Supplement: Table S3 — Statistics of 5-year relative survival rates by race and year of diagnosis, US. 1974–2001 (0.04 MB DOC) [file pone.0013696.s005.doc]

**Table S3:**  Statistics of 5-year relative survival rates () by race and year of diagnosis, US. 1974-2001

| Cancer  Site | Relative 5-Year Survival Rate () | | | | | | | | | No. of differential genes |
| --- | --- | --- | --- | --- | --- | --- | --- | --- | --- | --- |
|  | White | | | African American | | | All Race | | |  |
|  | 1974-76 | 1983-85 | 1995-2001 | 1974-76 | 1983-85 | 1995-2001 | 1974-76 | 1983-85 | 1995-2001 |  |
| Prostate | 68 | 76 | 100 | 58 | 64 | 97 | 67 | 75 | 100 | 118 |
| Breast | 75 | 79 | 90 | 63 | 64 | 76 | 75 | 78 | 88 | 294 |
| Kidney | 52 | 56 | 65 | 49 | 55 | 64 | 52 | 56 | 65 | 231 |
| Colon | 51 | 58 | 65 | 46 | 49 | 55 | 50 | 58 | 64 | 247 |
| Stomach | 15 | 16 | 21 | 16 | 19 | 23 | 15 | 17 | 23 | 311 |
| Lung & bronchus | 13 | 14 | 16 | 11 | 11 | 13 | 12 | 14 | 15 | 683 |
| Pancreas | 3 | 3 | 4 | 3 | 5 | 4 | 3 | 3 | 4 | 885 |
