# Supplementary material for: A Comparative Analysis of Gene-Expression Data of Multiple Cancer Types
Source: PLoS One. 2010 Oct 27;5(10):e13696. doi: 10.1371/journal.pone.0013696 (PMC2965162; doi:10.1371/journal.pone.0013696)
Supplement: Table S4 — The list of 85 genes that differentially expressed in more than 3 cancer type (0.19 MB DOC) [file pone.0013696.s006.doc]

**Table S4:** The list of 85 genes that differentially expressed in more than 3 cancer type

| **Gene ID** | **Direction of regulation** | | | | | | | | **Function** |
| --- | --- | --- | --- | --- | --- | --- | --- | --- | --- |
|  |  | Bre. | Con. | Kid. | Lun. | Pan. | Pro. | Sto. |  |
| ABCA8 | down | **↓** | **↓** | **↓** | **↓** |  |  | **↓** | ATP-dependent lipophilic drug transporter |
| ACADL | down | **↓** |  |  | **↓** | **↓** |  |  | NA |
| ADH1B | down | **↓** |  | **↓** | **↓** |  |  |  | NA |
| ADH1C | down | **↓** | **↓** |  |  |  |  | **↓** | NA |
| AGR2 | up | **↑** |  |  | **↑** | **↑** |  |  | NA |
| ALDH1A1 | down | **↓** | **↓** |  | **↓** |  |  | **↓** | Binds free retinal and cellular retinol-binding protein- bound retinal. Can convert/oxidize retinaldehyde to retinoic acid |
| ANLN | up |  | **↑** |  |  | **↑** |  | **↑** | Required for cytokinesis. Essential for the structural integrity of the cleavage furrow and for completion of cleavage furrow ingression. |
| AOC3 | down | **↓** | **↓** |  | **↓** |  |  |  | Cell adhesion protein that participates in lymphocyte recirculation by mediating the binding of lymphocytes to peripheral lymph node vascular endothelial cells in an L-selectin- independent fashion. Has a monoamine oxidase activity. |
| ASPM | up | **↑** |  |  | **↑** | **↑** |  |  | Probable role in mitotic spindle regulation and coordination of mitotic processes. May have a preferential role in regulating neurogenesis. |
| AURKA | up | **↑** | **↑** |  | **↑** |  | **↑** | **↑** | May play a role in cell cycle regulation during anaphase and/or telophase, in relation to the function of the centrosome/spindle pole region during chromosome segregation. May be involved in microtubule formation and/or stabilization. May play a key role during tumor development and progression. Phosphorylates ARHGEF2 and BORA. |
| CAV1 | down | **↓** |  |  | **↓** |  | **↓** |  | May act as a scaffolding protein within caveolar membranes. Interacts directly with G-protein alpha subunits and can functionally regulate their activity |
| CCL18 | up |  |  | **↑** |  | **↑** |  | **↑** | Chemotactic factor that attracts lymphocytes but not monocytes or granulocytes. May be involved in B-cell migration into B-cell follicles in lymph nodes. Attracts naive T-lymphocytes toward dendritic cells and activated macrophages in lymph nodes, has chemotactic activity for naive T-cells, CD4+ and CD8+ T-cells and thus may play a role in both humoral and cell-mediated immunity responses. |
| CD36 | down | **↓** |  |  | **↓** |  |  | **↓** | Seems to have numerous potential physiological functions. Binds to collagen, thrombospondin, anionic phospholipids and oxidized LDL. May function as a cell adhesion molecule. Directly mediates cytoadherence of Plasmodium falciparum parasitized erythrocytes. Binds long chain fatty acids and may function in the transport and/or as a regulator of fatty acid transport. |
| CDC2 | up | **↑** | **↑** |  | **↑** | **↑** |  | **↑** | Plays a key role in the control of the eukaryotic cell cycle. It is required in higher cells for entry into S-phase and mitosis. p34 is a component of the kinase complex that phosphorylates the repetitive C-terminus of RNA polymerase II. |
| CDH3 | up |  | **↑** |  | **↑** | **↑** |  |  | Cadherins are calcium dependent cell adhesion proteins. They preferentially interact with themselves in a homophilic manner in connecting cells; cadherins may thus contribute to the sorting of heterogeneous cell types. |
| CENPF | up | **↑** |  |  | **↑** | **↑** |  | **↑** | Probably required for kinetochore function, involved in chromosome segregation during mitosis. Interacts with retinoblastoma protein (RB), CENP-E and BUBR1. |
| CHRDL1 | down | **↓** |  |  | **↓** |  | **↓** |  | Antagonizes the function of BMP4 by binding to it and preventing its interaction with receptors. Alters the fate commitment of neural stem cells from gliogenesis to neurogenesis. Contributes to neuronal differentiation of neural stem cells in the brain by preventing the adoption of a glial fate. May play a crucial role in dorsoventral axis formation. May play a role in embyonic bone formation (By similarity). May also play an important role in regulating retinal angiogenesis trough modulation of BMP4 actions in endothelial cells. |
| CKS2 | up | **↑** |  |  |  | **↑** |  | **↑** | Binds to the catalytic subunit of the cyclin dependent kinases and is essential for their biological function. |
| CLDN4 | up |  |  |  | **↑** | **↑** |  | **↑** | Plays a major role in tight junction-specific obliteration of the intercellular space. |
| CLEC3B | down | **↓** |  |  | **↓** |  |  | **↓** | Tetranectin binds to plasminogen and to isolated kringle 4. May be involved in the packaging of molecules destined for exocytosis. |
| CNN1 | down |  | **↓** |  | **↓** |  | **↓** |  | Thin filament-associated protein that is implicated in the regulation and modulation of smooth muscle contraction. It is capable of binding to actin, calmodulin, troponin C and tropomyosin. The interaction of calponin with actin inhibits the actomyosin Mg-ATPase activity (By similarity). |
| COL11A1 | up | **↑** | **↑** |  | **↑** | **↑** |  |  | May play an important role in fibrillogenesis by controlling lateral growth of collagen II fibrils. |
| COL1A1 | up | **↑** |  |  | **↑** | **↑** |  |  | Type I collagen is a member of group I collagen (fibrillar forming collagen). |
| COX7A1 | down | **↓** |  |  | **↓** |  | **↓** |  | This protein is one of the nuclear-coded polypeptide chains of cytochrome c oxidase, the terminal oxidase in mitochondrial electron transport. |
| CXCL12 | down |  | **↓** |  | **↓** |  | **↓** |  | Chemoattractant active on T-lymphocytes, monocytes, but not neutrophils. SDF-1-beta(3-72) and SDF-1-alpha(3-67) show a reduced chemotactic activity. Binding to cell surface proteoglycans seems to inhibit formation of SDF-1-alpha(3-67) and thus to preserve activity on local sites. |
| DMD | down |  | **↓** |  |  | **↓** | **↓** |  | May play a role in anchoring the cytoskeleton to the plasma membrane. |
| DPT | down | **↓** | **↓** |  | **↓** |  | **↓** | **↓** | Seems to mediate adhesion by cell surface integrin binding. May serve as a communication link between the dermal fibroblast cell surface and its extracellular matrix environment. Enhances TGFB1 activity. Inhibits cell proliferation. Accelerates collagen fibril formation, and stabilizes collagen fibrils against low-temperature dissociation. |
| ECT2 | up |  | **↑** |  | **↑** | **↑** |  | **↑** | Binds highly specifically to RhoA, RhoC and Rac proteins, but does not appear to catalyze guanine nucleotide exchange. |
| ESM1 | up |  | **↑** |  |  | **↑** |  | **↑** | May have potent implications in lung endothelial cell- leukocyte interactions. |
| FABP4 | down | **↓** | **↓** |  | **↓** |  |  | **↓** | Lipid transport protein in adipocytes. Binds both long chain fatty acids and retinoic acid. Delivers long-chain fatty acids and retinoic acid to their cognate receptors in the nucleus. |
| FAM107A | down |  | **↓** |  | **↓** |  |  | **↓** | When transfected into cell lines in which it is not expressed, suppresses cell growth. May play a role in tumor development. |
| GPX3 | down | **↓** |  |  | **↓** |  |  | **↓** | Protects cells and enzymes from oxidative damage, by catalyzing the reduction of hydrogen peroxide, lipid peroxides and organic hydroperoxide, by glutathione. |
| GREM1 | up |  |  |  | **↑** | **↑** |  | **↑** | Cytokine that may play an important role during carcinogenesis and metanephric kidney organogenesis, as a BMP antagonist required for early limb outgrowth and patterning in maintaining the FGF4-SHH feedback loop. Down-regulates the BMP4 signaling in a dose-dependent manner. Acts as inhibitor of monocyte chemotaxis. |
| HBB | down | **↓** | **↓** |  | **↓** |  |  |  | LVV-hemorphin-7 potentiates the activity of bradykinin, causing a decrease in blood pressure. |
| HPGD | down |  |  | **↓** | **↓** |  |  | **↓** | Inactivation of prostaglandins. |
| HS3ST1 | up |  |  | **↑** | **↑** | **↑** |  |  | Rate limiting enzyme for synthesis of HSact. Performs the crucial step modification in the biosynthesis of anticoagulant heparan sulfate (HSact) that is to complete the structure of the antithrombin pentasaccharide binding site. |
| INHBA | up | **↑** |  |  |  | **↑** |  | **↑** | Inhibins and activins inhibit and activate, respectively, the secretion of follitropin by the pituitary gland. Inhibins/activins are involved in regulating a number of diverse functions such as hypothalamic and pituitary hormone secretion, gonadal hormone secretion, germ cell development and maturation, erythroid differentiation, insulin secretion, nerve cell survival, embryonic axial development or bone growth, depending on their subunit composition. Inhibins appear to oppose the functions of activins. |
| KLF4 | down | **↓** | **↓** |  | **↓** |  |  | **↓** | Transcription factor which acts as both an activator and repressor. Binds the CACCC core sequence. Binds to multiple sites in the 5'-flanking region of its own gene and can activate its own transcription. Required for establishing the barrier function of the skin and for postnatal maturation and maintenance of the ocular surface. Involved in the differentiation of epithelial cells and may also function in skeletal and kidney development. |
| KRT8 | up | **↑** |  |  | **↑** | **↑** |  |  | Together with KRT19, helps to link the contractile apparatus to dystrophin at the costameres of striated muscle. |
| LCN2 | up |  | **↑** |  | **↑** | **↑** |  |  | Transport of small lipophilic substances (Potential). |
| MAD2L1 | up |  | **↑** |  | **↑** | **↑** |  | **↑** | Required for the execution of the mitotic checkpoint which monitors the process of kinetochore-spindle attachment and delays the onset of anaphase when this process is not complete. It inhibits the activity of the anaphase promoting complex by sequestering CDC20 until all chromosomes are aligned at the metaphase plate. |
| MCM4 | up | **↑** |  |  | **↑** |  |  | **↑** | Involved in the control of DNA replication. |
| MDK | up | **↑** |  |  | **↑** | **↑** |  |  | Has heparin binding activity, and growth promoting activity. Involved in neointima formation after arterial injury, possibly by mediating leukocyte recruitment. Also involved in early fetal adrenal gland development (By similarity). |
| MELK | up | **↑** |  |  | **↑** | **↑** |  | **↑** | Phosphorylates ZNF622 and may contribute to its redirection to the nucleus. May be involved in the inhibition of spliceosome assembly during mitosis. |
| MME | down | **↓** |  | **↓** | **↓** |  |  |  | Thermolysin-like specificity, but is almost confined on acting on polypeptides of up to 30 amino acids. Biologically important in the destruction of opioid peptides such as Met- and Leu-enkephalins by cleavage of a Gly-Phe bond. Involved in the degradation of atrial natriuretic factor (ANF). |
| MMP1 | up |  | **↑** |  | **↑** | **↑** |  |  | Cleaves collagens of types I, II, and III at one site in the helical domain. Also cleaves collagens of types VII and X. In case of HIV infection, interacts and cleaves the secreted viral Tat protein, leading to a decrease in neuronal Tat's mediated neurotoxicity. |
| MMP11 | up |  |  |  | **↑** | **↑** |  | **↑** | May play an important role in the progression of epithelial malignancies. |
| MMP12 | up |  |  |  | **↑** | **↑** |  | **↑** | May be involved in tissue injury and remodeling. Has significant elastolytic activity. Can accept large and small amino acids at the P1' site, but has a preference for leucine. Aromatic or hydrophobic residues are preferred at the P1 site, with small hydrophobic residues (preferably alanine) occupying P3. |
| MMP7 | up |  | **↑** |  | **↑** | **↑** |  | **↑** | Degrades casein, gelatins of types I, III, IV, and V, and fibronectin. Activates procollagenase. |
| MMP9 | up |  |  |  | **↑** | **↑** |  | **↑** | May play an essential role in local proteolysis of the extracellular matrix and in leukocyte migration. Could play a role in bone osteoclastic resorption. Cleaves KiSS1 at a Gly-|-Leu bond. |
| MT1M | down |  |  |  | **↓** | **↓** |  | **↓** | Metallothioneins have a high content of cysteine residues that bind various heavy metals; these proteins are transcriptionally regulated by both heavy metals and glucocorticoids. |
| MT1X | down |  | **↓** |  |  |  | **↓** | **↓** | Metallothioneins have a high content of cysteine residues that bind various heavy metals; these proteins are transcriptionally regulated by both heavy metals and glucocorticoids. |
| MXRA5 | up |  |  |  | **↑** | **↑** |  | **↑** | NA |
| MYB | up | **↑** |  |  |  |  | **↑** | **↑** | Transcriptional activator; DNA-binding protein that specifically recognize the sequence 5'-YAAC[GT]G-3'. Plays an important role in the control of proliferation and differentiation of hematopoietic progenitor cells. |
| MYH11 | down |  | **↓** |  | **↓** |  | **↓** |  | Muscle contraction. |
| MYL9 | down | **↓** | **↓** |  | **↓** |  |  |  | Myosin regulatory subunit that plays an important role in regulation of both smooth muscle and nonmuscle cell contractile activity via its phosphorylation. Implicated in cytokinesis, receptor capping, and cell locomotion. |
| MYLK | down |  | **↓** |  | **↓** |  | **↓** |  | Calcium/calmodulin-dependent enzyme implicated in smooth muscle contraction via phosphorylation of myosin light chains (MLC). Implicated in the regulation of endothelial as well as vascular permeability. In the nervous system it has been shown to control the growth initiation of astrocytic processes in culture and to participate in transmitter release at synapses formed between cultured sympathetic ganglion cells. Critical participant in signaling sequences that result in fibroblast apoptosis. |
| NDC80 | up | **↑** |  |  | **↑** | **↑** |  |  | Acts as a component of the essential kinetochore- associated NDC80 complex, which is required for chromosome segregation and spindle checkpoint activity. Required for kinetochore integrity and the organization of stable microtubule binding sites in the outer plate of the kinetochore. |
| NUSAP1 | up |  |  |  | **↑** | **↑** |  | **↑** | Microtubule-associated protein with the capacity to bundle and stabilize microtubules (By similarity). May associate with chromosomes and promote the organization of mitotic spindle microtubules around them. |
| PAICS | up |  | **↑** |  | **↑** |  |  | **↑** | NA |
| PCK1 | down | **↓** | **↓** | **↓** |  |  |  |  | NA |
| PDK4 | down |  |  |  | **↓** | **↓** |  | **↓** | Inhibits the mitochondrial pyruvate dehydrogenase complex by phosphorylation of the E1 alpha subunit, thus contributing to the regulation of glucose metabolism. |
| PHLDA2 | up | **↑** |  |  | **↑** | **↑** |  |  | May play a role in regulating placenta growth. |
| PMAIP1 | up | **↑** | **↑** |  | **↑** | **↑** |  |  | Promotes activation of caspases and apoptosis. Promotes mitochondrial membrane changes and efflux of apoptogenic proteins from the mitochondria. Contributes to p53-dependent apoptosis after radiation exposure. Promotes proteasomal degradation of MCL1. Competes with BAK1 for binding to MCL1 and can displace BAK1 from its binding site on MCL1 (By similarity). Competes with BIM/BCL2L11 for binding to MCL1 and can displace BIM/BCL2L11 from its binding site on MCL1. |
| PRC1 | up | **↑** |  |  | **↑** | **↑** |  | **↑** | KIF4A translocates PRC1 to the plus ends of interdigitating spindle microtubules during the metaphase to anaphase transition, an essential step for the formation of an organized central spindle midzone and midbody and for successful cytokinesis. Required for KIF14 localization to the central spindle and midbody. Acts as a microtubule-binding and bundling protein both in vivo and vitro. May function as an in vivo cyclin- CDK substrate. |
| PTGER4 | down |  | **↓** |  | **↓** | **↓** |  |  | Receptor for prostaglandin E2 (PGE2). The activity of this receptor is mediated by G(s) proteins that stimulate adenylate cyclase. Has a relaxing effect on smooth muscle. May play an important role in regulating renal hemodynamics, intestinal epithelial transport, adrenal aldosterone secretion, and uterine function. |
| PTRF | down | **↓** |  |  | **↓** |  | **↓** |  | Termination of transcription by RNA polymerase I involves pausing of transcription by TTF1, and the dissociation of the transcription complex, releasing pre-rRNA and RNA polymerase I from the template. PTRF is required for dissociation of the ternary transcription complex (By similarity). |
| PTTG1 | up |  | **↑** |  |  | **↑** |  | **↑** | Regulatory protein, which plays a central role in chromosome stability, in the p53/TP53 pathway, and DNA repair. Probably acts by blocking the action of key proteins. During the mitosis, it blocks Separase/ESPL1 function, preventing the proteolysis of the cohesin complex and the subsequent segregation of the chromosomes. At the onset of anaphase, it is ubiquitinated, conducting to its destruction and to the liberation of ESPL1. Its function is however not limited to a blocking activity, since it is required to activate ESPL1. Negatively regulates the transcriptional activity and related apoptosis activity of TP53. The negative regulation of TP53 may explain the strong transforming capability of the protein when it is overexpressed. May also play a role in DNA repair via its interaction with Ku, possibly by connecting DNA damage-response pathways with sister chromatid separation. |
| RNASE1 | down |  | **↓** |  |  | **↓** |  | **↓** | Endonuclease that catalyzes the cleavage of RNA on the 3' side of pyrimidine nucleotides. Acts on single stranded and double stranded RNA. |
| RRM2 | up | **↑** |  |  | **↑** | **↑** |  | **↑** | Provides the precursors necessary for DNA synthesis. Catalyzes the biosynthesis of deoxyribonucleotides from the corresponding ribonucleotides. Inhibits Wnt signaling. |
| S100P | up | **↑** |  |  | **↑** | **↑** |  |  | NA |
| SDPR | down | **↓** | **↓** |  | **↓** |  |  |  | May play a role in targeting PRKCA to caveolae (By similarity). |
| SFN | up |  |  | **↑** | **↑** | **↑** |  |  | p53-regulated inhibitor of G2/M progression. |
| SOX4 | up |  |  |  | **↑** | **↑** |  | **↑** | Transcriptional activator that binds with high affinity to the T-cell enhancer motif 5'-AACAAAG-3' motif. |
| SPARCL1 | down |  | **↓** |  | **↓** |  | **↓** |  | NA |
| SULF1 | up |  |  |  | **↑** | **↑** |  | **↑** | Exhibits arylsulfatase activity and highly specific endoglucosamine-6-sulfatase activity. It can remove sulfate from the C-6 position of glucosamine within specific subregions of intact heparin. Diminishes HSPG (heparan sulfate proteoglycans) sulfation, inhibits signaling by heparin-dependent growth factors, diminishes proliferation, and facilitates apoptosis in response to exogenous stimulation. |
| TGFBI | up |  |  | **↑** |  | **↑** |  | **↑** | Binds to type I, II, and IV collagens. This adhesion protein may play an important role in cell-collagen interactions. In cartilage, may be involved in endochondral bone formation. |
| TGFBR3 | down | **↓** | **↓** |  | **↓** |  |  |  | Binds to TGF-beta. Could be involved in capturing and retaining TGF-beta for presentation to the signaling receptors. |
| THBS2 | up |  |  | **↑** | **↑** | **↑** |  |  | Adhesive glycoprotein that mediates cell-to-cell and cell-to-matrix interactions. Can bind to fibrinogen, fibronectin, laminin and type V collagen. |
| TOP2A | up | **↑** | **↑** |  | **↑** | **↑** |  | **↑** | Control of topological states of DNA by transient breakage and subsequent rejoining of DNA strands. Topoisomerase II makes double-strand breaks. |
| TOX3 | up | **↑** |  |  | **↑** | **↑** |  |  | NA |
| TPX2 | up |  |  |  | **↑** |  | **↑** | **↑** | NA |
| TTK | up |  | **↑** |  | **↑** |  | **↑** | **↑** | Phosphorylates proteins on serine, threonine, and tyrosine. Probably associated with cell proliferation. |
| UBE2C | up |  | **↑** |  | **↑** |  |  | **↑** | Catalyzes the covalent attachment of ubiquitin to other proteins. Required for the destruction of mitotic cyclins. |

“↑” indicates that a gene is up-regulated in the corresponding cancer type while “↓” indicates that a gene is down-regulated
