# Supplementary material for: A Comparative Analysis of Gene-Expression Data of Multiple Cancer Types
Source: PLoS One. 2010 Oct 27;5(10):e13696. doi: 10.1371/journal.pone.0013696 (PMC2965162; doi:10.1371/journal.pone.0013696)
Supplement: Table S5 — Enriched pathways by differentially expressed genes in different cancer types (enrichment P-value cutoff = 0.05) (0.09 MB DOC) [file pone.0013696.s007.doc]

**Table S5: Enriched pathways by differentially expressed genes in different cancer types (enrichment P-value cutoff = 0.05)**

| Pathways | COUNT | BREAST | COLON | KIDNEY | LUNG | PANCREASE | PROSTATE | STOMACH |
| --- | --- | --- | --- | --- | --- | --- | --- | --- |
| Complement and coagulation cascades | 4 |  | X | X | X | X |  |  |
| ECM-receptor interaction | 4 | X |  |  | X | X |  | X |
| Focal adhesion | 4 | X |  |  | X | X | X |  |
| Cell Communication | 4 | X |  |  | X | X | X |  |
| Cell adhesion molecules (CAMs) | 3 |  |  |  | X | X |  | X |
| PPAR signaling pathway | 3 | X | X | X |  |  |  |  |
| Glycine, serine and threonine metabolism | 3 |  | X | X |  | X |  |  |
| p53 signaling pathway | 2 |  |  |  | X | X |  |  |
| Cell cycle | 2 |  |  |  | X |  |  | X |
| Glycolysis / Gluconeogenesis | 2 |  |  | X |  | X |  |  |
| Platelet Amyloid Precursor Protein Pathway | 2 |  |  | X |  | X |  |  |
| PKC-catalyzed phosphorylation of inhibitory phosphoprotein of myosin phosphatase | 2 |  | X |  |  |  | X |  |
| Fibrinolysis Pathway | 2 |  |  | X |  | X |  |  |
| Eicosanoid Metabolism | 2 |  | X |  |  | X |  |  |
| RBphosphoE2F | 2 | X |  |  | X |  |  |  |
| Adipocytokine signaling pathway | 1 | X |  |  |  |  |  |  |
| Small cell lung cancer | 1 |  |  |  |  | X |  |  |
| Hematopoietic cell lineage | 1 |  |  |  |  | X |  |  |
| TGF-beta signaling pathway | 1 |  |  |  | X |  |  |  |
| Cytokine-cytokine receptor interaction | 1 |  |  |  |  | X |  |  |
| Calcium signaling pathway | 1 |  |  |  |  |  | X |  |
| ABC transporters – General | 1 |  | X |  |  |  |  |  |
| Metabolism of xenobiotics by cytochrome P450 | 1 |  |  |  |  |  |  | X |
| Nitrogen metabolism | 1 |  |  |  |  |  |  | X |
| 3-Chloroacrylic acid degradation | 1 | X |  |  |  |  |  |  |
| Propanoate metabolism | 1 | X |  |  |  |  |  |  |
| Pyruvate metabolism | 1 | X |  |  |  |  |  |  |
| Linoleic acid metabolism | 1 |  |  |  |  |  |  | X |
| Arachidonic acid metabolism | 1 |  |  |  |  |  |  | X |
| Glycerophospholipid metabolism | 1 | X |  |  |  |  |  |  |
| Glycerolipid metabolism | 1 | X |  |  |  |  |  |  |
| O-Glycan biosynthesis | 1 |  |  |  |  | X |  |  |
| Tryptophan metabolism | 1 | X |  |  |  |  |  |  |
| Tyrosine metabolism | 1 |  |  | X |  |  |  |  |
| Histidine metabolism | 1 | X |  |  |  |  |  |  |
| Arginine and proline metabolism | 1 |  |  | X |  |  |  |  |
| Urea cycle and metabolism of amino groups | 1 | X |  |  |  |  |  |  |
| Bile acid biosynthesis | 1 | X |  |  |  |  |  |  |
| Visceral Fat Deposits and the Metabolic Syndrome | 1 | X |  |  |  |  |  |  |
| Vitamin C in the Brain | 1 |  |  |  |  | X |  |  |
| Inhibition of Matrix Metalloproteinases | 1 |  |  |  | X |  |  |  |
| IGF-1 Receptor and Longevity | 1 | X |  |  |  |  |  |  |
| Low-density lipoprotein (LDL) pathway during atherogenesis | 1 |  |  |  | X |  |  |  |
| Classical Complement Pathway | 1 |  |  | X |  |  |  |  |
| Pertussis toxin-insensitive CCR5 Signaling in Macrophage | 1 |  | X |  |  |  |  |  |
| Chemokine_families | 1 |  | X |  |  |  |  |  |
