# Supplementary material for: A Comparative Analysis of Gene-Expression Data of Multiple Cancer Types
Source: PLoS One. 2010 Oct 27;5(10):e13696. doi: 10.1371/journal.pone.0013696 (PMC2965162; doi:10.1371/journal.pone.0013696)
Supplement: Table S6 — The top 3-gene and 4-gene discriminators for multiple cancer type types (0.06 MB DOC) [file pone.0013696.s008.doc]

**Table S6a**: The top 3-gene discriminators for multiple cancer type types (accuracy by percentage)

| **Count** | **Markers** | **Breast** | | | **Colon** | | | **Kidney** | | | **Lung** | | | **Pancreas** | | | **Prostate** | | | **Stomach** | | |
| --- | --- | --- | --- | --- | --- | --- | --- | --- | --- | --- | --- | --- | --- | --- | --- | --- | --- | --- | --- | --- | --- | --- |
|  |  | **train** | **test** | **test2** | **train** | **test** | **test2** | **train** | **test** | **test2** | **train** | **test** | **test2** | **train** | **test** | **test2** | **train** | **test** | **test2** | **train** | **test** | **test2** |
| 4 | ABCA8+ALDH1A1+DPT | 75.9 | 96.6 | 92.2 | 93.8 | 97.9 | 81.0 | _ | _ | _ | 92.6 | 96.3 | 92.3 | _ | _ | _ | _ | _ | _ | 75.4 | 91.3 | 70.4 |
| 4 | ABCA8+AURKA+DPT | 77.6 | 96.6 | 99.2 | 95.8 | 100 | miss | _ | _ | _ | 92.6 | 94.4 | 89.7 | _ | _ | _ | _ | _ | _ | 76.8 | 91.3 | 70.4 |
| 4 | ALDH1A1+FABP4+TOP2A | 79.3 | 93.1 | 92.2 | 87.5 | 85.4 | 75.3 | _ | _ | _ | 96.3 | 77.8 | 71.8 | _ | _ | _ | _ | _ | _ | 76.8 | 76.8 | 81.5 |

**Table S6b:** The top 4-gene discriminators for multiple cancer types (accuracy by percentage)

| **Count** | **Markers** | **Breast** | | | **Colon** | | | **Kidney** | | | **Lung** | | | **Pancreas** | | | **Prostate** | | | **Stomach** | | |
| --- | --- | --- | --- | --- | --- | --- | --- | --- | --- | --- | --- | --- | --- | --- | --- | --- | --- | --- | --- | --- | --- | --- |
|  |  | **train** | **test** | **test2** | **train** | **test** | **test2** | **train** | **test** | **test2** | **train** | **test** | **test2** | **train** | **test** | **test2** | **train** | **test** | **test2** | **train** | **test** | **test2** |
| 4 | ABCA8+CDC2+KLF4+TOP2A | 79.3 | 94.8 | 98.4 | 93.8 | 100 | 83.3 | _ | _ | _ | 94.4 | 98.1 | 92.3 | _ | _ | _ | _ | _ | _ | 88.4 | 94.2 | 77.7 |
| 4 | ABCA8+FABP4+KLF4+TOP2A | 79.3 | 96.6 | 94.6 | 89.6 | 100 | 83.3 | _ | _ | _ | 96.3 | 98.1 | 94.9 | _ | _ | _ | _ | _ | _ | 87.0 | 89.9 | 81.5 |
| 4 | ALDH1A1+FABP4+KLF4+TOP2A | 79.3 | 94.8 | 95.3 | 89.6 | 97.9 | 78.6 | _ | _ | _ | 96.3 | 96.3 | 94.9 | _ | _ | _ | _ | _ | _ | 85.5 | 91.3 | 81.5 |
| 4 | CDC2+COL11A1+PMAIP1+TOP2A | 79.3 | 100 | 98.4 | 93.8 | 100 | 78.6 | _ | _ | _ | 90.7 | 90.7 | 89.7 | 90.4 | 86.5 | 82.4 | _ | _ | _ | _ | _ | _ |
| 4 | DPT+FABP4+KLF4+TOP2A | 79.3 | 94.8 | 92.2 | 91.7 | 100 | 85.7 | _ | _ | _ | 98.1 | 96.3 | 97.4 | _ | _ | _ | _ | _ | _ | 85.5 | 91.3 | 81.5 |
