# Supplementary figures and images for: A Comparative Analysis of Gene-Expression Data of Multiple Cancer Types
Source: PLoS One. 2010 Oct 27;5(10):e13696. doi: 10.1371/journal.pone.0013696 (PMC2965162; doi:10.1371/journal.pone.0013696)

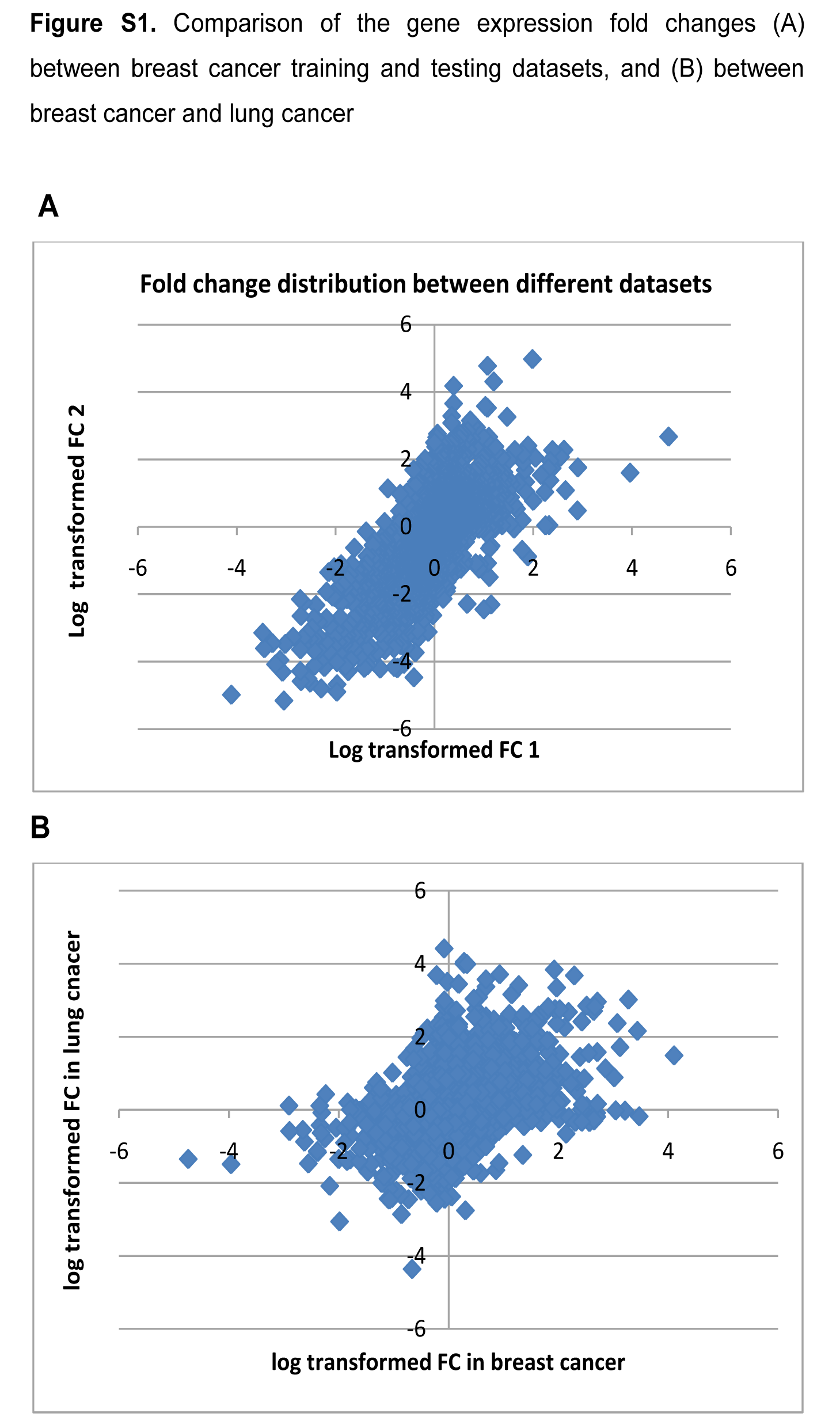

Supplement: Figure S1 — Comparison of the gene expression fold changes (A) between breast cancer training and testing datasets, and (B) between breast cancer and lung cancer (0.30 MB TIF) [file pone.0013696.s009.tif]
